# Effect of Carbohydrate Formulas on Instrumental and Sensory Parameters in Dry-Fermented Iberian Pork Sausages

**DOI:** 10.3390/foods14020248

**Published:** 2025-01-14

**Authors:** Maite Sánchez-Giraldo, Montserrat Vioque-Amor, Rafael Gómez-Díaz, Ignacio Clemente-López, Manuel Ángel Amaro-López, Carmen Avilés-Ramírez

**Affiliations:** 1 Research Group AGR-120, Department of Food Science and Technology, University of Cordoba, Ctra. Madrid-Cadiz km 396, 14071 Cordoba, Spain; t32sagim@uco.es (M.S.-G.); bt1viamm@uco.es (M.V.-A.); bt1godir@uco.es (R.G.-D.); bt1amlom@uco.es (M.Á.A.-L.); 2 DOSCADESA 2000 S.L., Molina de Segura, 30500 Murcia, Spain

**Keywords:** dry-fermented sausages, dextrose, dextrin, glucose syrup, Iberian pork

## Abstract

Dry-fermented sausages are appreciated all over the world for their sensory characteristics. Carbohydrates (sugars) are added during the production process, the type and quantity of which directly affect the quality of this product. However, there are few data on the role of sugars on instrumental and sensory parameters of sausages derived from Iberian pork. The objective was to determine the effect of different carbohydrate formulas during the ripening time on the quality of dry-fermented Iberian pork sausages. Five batches were formulated with different combinations and concentrations of carbohydrates (dextrose, dextrin and glucose syrup), making 16 sausages per batch on which to perform repeated measurements throughout the ripening process. Physicochemical characteristics, instrumental colour, textural parameters and sensory attributes were analysed. The C− batch (without any carbohydrate addition) showed unstable pH evolution and high b* values throughout the ripening process and the lowest instrumental texture values (for hardness, springiness and chewiness). The D10d5 batch also presented an unstable pH evolution but low a* values, and it was the highest rated by consumers for sensory texture attributes. This batch, made with dextrose (in higher proportion) and dextrin, differed from the other Iberian pork sausages, suggesting that this carbohydrate formula may be more appreciated by Mediterranean consumers.

## 1. Introduction

Dry-fermented sausages are defined, according to current regulations, as those that undergo a salting and curing–ripening process in order to give them their own organoleptic characteristics and stability at room temperature [1]. The traditional preparation of these products has been by mixing lean meat and minced fat with different ingredients (such as sugars, aromatic herbs, spices and starter cultures) or additives (curing salts, antioxidants, etc.). Afterwards, the mixture is stuffed into different types of casings, where the fermentation is carried out. Finally, they are left to dry and, after ripening, are ready for consumption. 

These products are spread widely throughout the world and are part of the culture of different geographical regions, as they are highly appreciated for their sensory characteristics [2]. Even though the meat sector has developed alternatives to cured meat [3], the general public have not excluded these cured meats from their diet [4]. In addition, according to the latest Spanish Ministry of Agriculture, Fisheries and Food (MAPA) report [5], there has been an increase in the consumption of Iberian pork products. These artisanal products have a strong traditional component linked to the agro-sylvo-pastoral system of the “Dehesa”, which is typical of the Mediterranean area [6].

It is therefore essential to explore in depth the different instrumental and sensory aspects of these products to develop new combinations that could mimic the characteristics of the current ones. Sensory properties are attractive elements for consumers, encouraging them to purchase, consume and enjoy these “ready-to-eat” (RTE) foods [7].

The carbohydrate content of fresh post-rigor meat is 0.08–0.1%, which is insufficient to produce significant amounts of lactic acid, which is responsible for the protein coagulation during the fermentation of these products [8]. Therefore, during the production process, various sugars are added as a fermentation substrate for lactic acid bacteria (up to 2%, although 0.3–0.8% sucrose or glucose is usually sufficient). The type and amount of sugar will directly affect the pH and the time needed to decrease the sugar content as much as possible during the drying stage, resulting in the formation of a viscous–colloidal structure that gives the final product a firm texture [9]. 

To help form this gel, the proteins (which account for over 20% of the total weight of the meat) are denatured and arranged in an ordered and stable three-dimensional network, which is probably accentuated due to the presence of carbohydrates in the medium [10]. The ability of proteins to form this structure is decisive in the texture change, as gel properties are directly related to the textural properties of dry-fermented sausages [11].

Proteins are also involved in the formation of colour in dry-fermented sausages during the production process. In the sausage production process, and after several reactions, myoglobin and oxymyoglobin proteins are transformed into nitrosylmyoglobin, which has a dark red colour and gives the product its typical colour and stability [8]. 

Hughes et al. [12] pointed out that, although the texture and colour of these products are determined by protein composition and proteolysis, they could be affected by variation in the carbohydrates. In addition, these carbohydrates are involved in reactions that contribute to developing the aroma.

González-Fernández et al. [13] evaluated the influence of glucose concentration on instrumental and sensory parameters in dry-fermented sausages in Spain. However, there has been little research published to date on the effect of sugars on the texture, colour and sensory development of dry-fermented sausages from the Mediterranean area, specifically those derived from Iberian pork, nor has the influence of the different nature of the carbohydrates present in these products been described, which is of particular interest for the additives industry and the Iberian pork meat products sector.

With this background, our objective was to determine the effect of different carbohydrate formulas on the quality of dry-fermented Iberian pork sausages during the ripening process in order to select the most suitable product for the industry which appeals most to the consumer.

## 2. Materials and Methods

### 2.1. Experimental Design and Preparation of Dry-Fermented Sausages

The dry-fermented sausages were produced at a local manufacturer following the manufacturer's own process. To prepare the common mixture for manufacturing the dry-fermented sausages, the following ingredients were used: minced lean Iberian pork (65%) and minced Iberian pork belly (30.71%) obtained from local producers. The lean and the pork belly were ground through a 6 mm diameter mincing plate. Common batter also contained NaCl (2.31%), sodium ascorbate (0.05%), sodium nitrite (0.015%), potassium nitrate (0.015%) and a common flavouring base (0.4%) composed of spices (black pepper, nutmeg, garlic and a natural flavour-enhancing aroma). A negative control (C−) with no carbohydrates and five different treatments corresponding to the five experimental formulas with varied carbohydrates (dextrose, dextrin and glucose syrup) were prepared using 15 g/kg of dextrose (D15), 10 g/kg of dextrose + 5 g/kg of dextrin (D10d5), 5 g/kg of dextrose + 10 g/kg of dextrin (D5d10), 5 g/kg of dextrose + 5 g/kg of dextrin + 5 g/kg of glucose syrup (D5d5g5) and 15 g/kg of dextrin (d15). Flora Italia LC and BLC-48 starter cultures were added to all the batches (*L. sakei* sucrose + strain and *S. carnosus* spp.). The meat masses used presented mean values of 28.3 ± 3.92% fat, 13.60 ± 1.09% protein and 3.01 ± 0.23% ash. 

Five batches of approximately 10 kg each were produced, making a total of 16 dry-fermented sausages per batch. The meat mixtures were stuffed into 40 mm diameter synthetic collagen casings to obtain a sausage with an initial weight of between 230 and 250 g when fresh, reaching a weight of around 170 g when cured. 

The fermentation and ripening processes were carried out between 6 and 13 °C, with a relative humidity (RH) of 76 to 88% until water activity (a_W_) values below 0.920 and a minimum weight loss of 30% were obtained. To monitor the ripening process, controls were carried out on the initial mixture (day 0) in duplicate and on two sausages at 1, 2, 3, 7, 14, 21 and 28 days post-processing (on days 0, 2 and 3, only pH and a_W_). The parameters considered in the study were pH, a_W_, moisture, weight loss, colour and texture (the last was only measured by the end of the ripening process, day 28). All measurements were made in triplicate.

### 2.2. Measurement of pH, a_W_, Moisture Content and Weight Loss

To study the effect of dextrose, dextrin and glucose syrup on basic instrumental parameters of dry-fermented sausages, pH, a_W_, moisture and weight loss were determined. To measure pH, the penetration probe of a pH-meter (model HI 2020 with a digital electrode model HI 11310 integrated with a temperature sensor, Hanna Instruments S.L., Gipuzkoa, Spain) was introduced inside the mass of each sausage sample. The pH electrode was recalibrated at room temperature every five samples, using two standard buffer solutions at pH 4.0 and 7.0 (Hach, Loveland, CO, USA), and it was rinsed between measurements. Water activity was measured using a dewpoint hygrometer (model AquaLab 4, Decagon Inc., Pullman, WA, USA), with an accuracy of 0.003 units, and measurements between 0.03 and 1.00 a_W_ units, following the manufacturer's instructions (the calibration of the equipment was performed with saturated solutions of known a_W_). Moisture was measured by drying 10 g of the sample in an oven until constant weight, at 105 ± 1 °C, following the procedure described in the AOAC reference method 935.29 [14]. Weight loss was measured in two sausages per batch and replicated. Each sample was weighed during the ripening process (days 0, 1, 7, 14, 21 and 28). Weight loss was calculated using the following arithmetical expression:

%weight loss = ((W_0_ − W_d_)/W_0_) × 100

where W_0_ is weight of the product on day 1 of processing (g) and W_d_ is weight on the sampling day during the ripening process (g).

### 2.3. Measurement of Colour 

Instrumental colour was measured to evaluate the influence of the carbohydrates added on the visual colour of the samples. This was carried out on the surface of a slice of 4 mm using a colorimeter (Konica Minolta CR-400, Minolta Co., Osaka, Japan) with a D65 standard illuminant, an 8° visual angle and an 8 mm measurement aperture, according to the L* (lightness), a* (redness) and b* (yellowness) system [15], and the mean value was reported. The instrument was standardised, with respect to the whole calibration plate, before measurements were taken. The correction parameters of the CR-A43 white calibration plate were Y = 86.6, x = 0.3187 and y = 0.336.

### 2.4. Texture Profile Analysis 

For texture profile analysis (TPA), a double compression cycle test was applied on three cylinders of sausage of 1 cm in height and 3 cm in diameter, with a space of 5 s elapsing from the end of the first cycle to the beginning of the second, up to a compression of 50% of the original portion height. A P/25 aluminium cylindrical probe (25 mm diameter) was used at room temperature (23 °C) coupled to a texture analyser (model TA-XT plus, Texture Technologies Corp. and Stable Micro Systems, Hamilton, MA, USA) with the Texture Exponent 32 computer software (version 1.0.0.68, Stable Micro Systems, Surrey, UK). Forced deformation curves were obtained with a 25 kg load cell applied at a crosshead speed of 2 mm/s. The following parameters were measured: hardness (N), as the maximum force required to compress the sample; springiness, as the ability of the sample to recover its original shape after the cessation of the deforming force; adhesiveness (N × s), as the work required to overcome the attracting forces between the sample surface and the probe surface; cohesiveness, as the ratio of the area under the second compression and first compression curves; and finally, chewiness (N), as the product of springiness, cohesiveness and hardness. 

### 2.5. Sensory Analysis

At the end of the process, the dry-fermented sausages were tested by a panel of 100 consumers. The analysis was performed in a tasting room equipped with individual booths. The casings were removed, and the dry-fermented sausages were cut into slices 4 mm thick with an automatic slicer (model CF1007N, Jata, Guipuzcoa, Spain) and served at room temperature on white plastic plates. The consumers were provided with water and breadsticks to cleanse the palate between samples. The consumers tasted three samples, identified by random codes following a balanced complete block experimental design. For each sample, the consumers gave their scores for lean colour, fat colour, texture and overall quality, using a nine-box hedonic scale (1, dislike extremely–9, like extremely).

### 2.6. Statistical Analysis

To find the effect of the ripening time, the experimental formula used and the interaction of both on pH, a_W_, moisture, weight loss and colour indexes, a factorial ANOVA was performed using the following statistical model:

Y_ij_ = μ + D_i_ + B_j_ + (D × B)_ij_ + e_ij_

where Y_ij_ = determined parameter, μ = least squares mean value, D_i_ = fixed effect of ripening day (i = 1: day 1, i = 2: day 7, i = 3: day 14, i = 4: day 21, i = 5: day 28), B_j_ = fixed effect of formulation (j = 1: Control, j = 2: D15, j = 3: D10d5, j = 4: D5d10, j = 5: D5d5g5, j = 6: d15), e_ij_ = random residual. Subsequently, a multiple comparison analysis of means was carried out using Tukey’s HSD post hoc test with *p* < 0.05. 

A one-way ANOVA was used to assess the effect of the experimental formula on the TPA variables and sensory analysis. The relationship between the instrumental parameters and sensory attributes was examined using simple correlation coefficients. All the analyses were carried out using the STATISTICA software, version 12, StatSoft, Inc., Tulsa, OK, USA (2014).

## 3. Results

### 3.1. Physico-Chemical Measurements

The pH values of the dry-fermented sausages during the ripening process are shown in Table 1. The mean pH value was 6.06, which diminished until reaching a value that ranged between 5.58 and 5.04 for batch C− and batch D5d10, respectively, at the end of ripening. The decrease in pH values was very pronounced from day 4 to 7 of the ripening period (*p* < 0.001) in all batches, and it then stabilised without significant fluctuations from day 7 until day 28 (See Figure S1 in Supplementary Materials), except for batches C− and D10d5, which presented a more unstable evolution. The evolution of pH during ripening was significantly affected by the carbohydrate formula (*p* < 0.001). Not surprisingly, D10d5 reached the lowest pH value (4.98) on day 7. The pH values of the D5d5g5 batch were particularly low throughout the ripening process.

Water activity mean values are shown in Table 1. The water activity decreased almost constantly during the ripening period in all batches. Values ranged between 0.986 from the D15 batch on day 0 to 0.877 from the C− batch on day 28, with a significant decrease (*p* < 0.001) in all batches starting from day 7 (See Figure S2 in Supplementary Materials). Only on day 14 were significant differences (*p* < 0.05) observed in a_W_ between batches, showing two decreasing patterns: the one followed by batches D15, D10d5 and d15, which was more pronounced, and the pattern of batches C−, D5d10 and D5d5g5, which showed a more subtle decline in a_W_.

Moisture and weight loss values are presented in Table 2. A significant, gradual decrease (*p* < 0.001) in moisture content was observed in all the batches during the ripening period. Mean values ranged from 57.5% at the beginning of the ripening to 29.1% at the end. No significant differences (*p* > 0.05) were observed in moisture among batches at any ripening time (See Figure S3 in Supplementary Materials). 

Weight loss values increased during the ripening period. Mean values ranged from 1.89% on day 1 to 39.1% on day 28. The increase in weight loss values was particularly marked from day 1 to day 7, where significant differences (*p* < 0.01) were detected among batches (See Figure S3 from the Supplementary Materials). Weight loss values were used to establish the end of ripening, which occurred when they reached 40%.

### 3.2. Instrumental Colour

As can be seen in Table 3, L*, a* and b* values were significantly affected by both ripening time and batch. 

As for L* values, a downward trend was observed during the ripening process in almost all batches, except for C− and D5d10. However, only batches D15 and D10d5 presented significant differences (*p* < 0.001) among ripening days with the lowest values at the end of the process. There were significant differences among batches on days 1, 7 and 28. D15 and D10d5 presented the lowest L* value by the end of the ripening but showed a very different trend. While the L* value for the D10d5 batch showed the highest value on day 1 (48.47) and, from there, experienced a sharp, continuous decline, the D15 batch presented a fluctuating decrease throughout the entire process. The C− batch presented the highest L* value at the end of the process. 

Regarding the a* coordinate, heterogeneous results were observed in all the batches throughout ripening. Batches D10d5 and D5d10 presented significant differences in a* values over the different days. The most reddish values were detected on days 14 and 21, respectively. Only on day 28 significant differences among batches were detected. C− batch presented the highest value, while D10d5 and D5d10 presented the lowest. 

Concerning the b* value, a significant (*p* < 0.001) downward trend was observed throughout the ripening process in all batches except for C−. The differences between batches were significant on days 7, 21 and 28. Again, the C− batch presented the highest b* value at the end of the ripening process, while batches D15 and D5d10 showed the lowest.

In addition, a trend towards significance (*p* < 0.1) was observed in the positive correlation (r = 0.7489) between parameters a* and b* (Table S1). The yellowness index also showed a negative correlation with texture parameters such as hardness (r = −0.8715) and chewiness (r = −0.9059). 

### 3.3. Texture Profile Analysis 

Table 4 shows the mean values for each of the textural variables analysed for dry-fermented sausages with different compositions. 

Batch C− presented the lowest values for hardness, springiness and chewiness (*p* < 0.01), in contrast to the other batches, in which no significant differences were observed. No significant differences were found for adhesiveness and cohesiveness across batches, with a mean value of −0.97 for the former variable and 0.68 for the latter. 

### 3.4. Sensory Analysis

**Figure 1:**
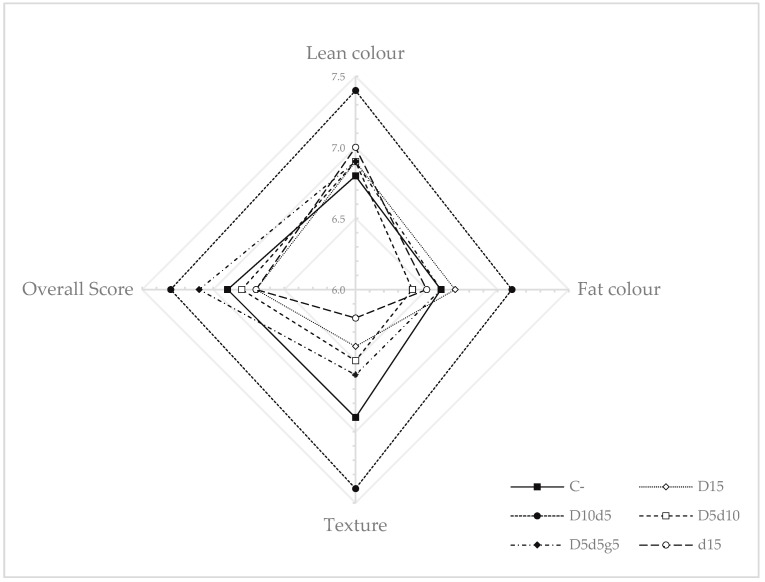
Sensory parameters (lean colour, fat colour, texture and overall score) in dry-fermented sausages manufactured with different carbohydrate formulas. C− = negative control, D15 = 15 g/kg dextrose, D10d5 = 10 g/kg dextrose + 5 g/kg dextrin, D5d10 = 5 g/kg dextrose + 10 g/kg dextrin, D5d5g5 =5 g/kg dextrose + 5 g/kg dextrin + 5 g/kg glucose syrup, d15 = 15 g/kg dextrin.

The results obtained from the sensory analysis are shown in Figure 1. No significant differences (*p* > 0.05) were observed across batches for the sensory attributes evaluated. However, a trend toward significance (*p* < 0.1) was observed for the attribute texture, with D10d5 being the highest rated by consumers and batch d15 the least valued. This attribute presented a positive correlation (*p* < 0.001) with the overall score (r = 0.9793) (Table S1). In addition, lean colour showed positive correlations (*p* < 0.05) with fat colour (r = 0.8918), texture (r = 0.8293) and overall score (r = 0.8495).

## 4. Discussion

### 4.1. Physico-Chemical Determinations

The pH values obtained in the finished product of this study were in the ranges previously described by different authors for products such as fuet and salami [12,16,17]. 

The slow pH drop observed from the beginning of the process until day 4 may have been due to the low temperatures used in the ripening of the Iberian sausages, which may have resulted in an extended lag phase at the beginning of the fermentation, since higher temperatures are needed to promote fermentation. However, the process of fermentation may have been affected by the temperature due to the use of Iberian pig belly fat, because the saturated fatty acids (SFA) contribute to the increase in fat firmness due of its high melting point [18], and belly fat of this breed is reported to present a low SFA content [19]. The slow, steady decline in pH during the fermentation of the product nevertheless seems beneficial, as it leads to a partial solubilization of actomyosin, which helps bind the particles together. As the process continues, the solubilized proteins gradually coagulate, resulting in a firm gel [20]. The drop in pH values detected between days 4 and 7 of the ripening process was due to the fermentation of the sugars by LAB, with the consequent production of lactic acid [21]. 

The pH stability detected from day 7 onwards might be the result of the slowdown in LAB growth due to the depletion of sugar stocks. The mean pH value increased slightly in the later stages of the ripening due to the reaction of lactic acid with the amino groups resulting from peptide degradation carried out by endogenous proteases and those from the starter culture [22]. Final pH values such as those obtained in this experiment guarantee the predominance of lactic microflora over possible pathogenic and spoilage microorganisms [23]. These pH values, approaching the isoelectric point of the myofibrillar component in the muscle, reduce the water-holding capacity (WHC) of the sausage due to the formation of a gel consisting of coagulated solubilized proteins. This not only promotes dehydration but also reinforces the structural cohesiveness.

The level of acidification depends on intrinsic and extrinsic factors that modulate the growth of microorganisms, in particular the activity of starter cultures and the addition of sugars. Batch C−, with no added sugars, presented higher pH values than the rest of the batches. González-Fernández et al. [13] observed that the concentration of glucose in the formula significantly affected the decrease in pH value during ripening, with pH values of sausages with low amounts of glucose dropping more slowly than those sausages with higher sugar concentration. Cured meat products with a_W_ values lower than 0.90 are considered self-stable meat products [24], and the a_W_ values of the dry-fermented sausages from all the batches in our study were within the range of values established as safe and stable for similar cured Iberian products [25]. In addition, the a_W_ value is proportional to the degree of dehydration in water-rich foods, such as fresh meat, although the addition of hygroscopic substances such as salt, sugar and others can reduce the diffusion of water from the sausage. In our study, the results obtained for moisture coincide with those described by Gómez et al. [26] and Mora-Gallego et al. [27] but were slightly lower than those reported by Corral et al. [28]. Nevertheless, the amount and type of sugar added to the sausages impact the WHC and, therefore, the textural attributes of the products, although the specific conditions used during the drying stage must also be considered [29]. Both Qiu et al. [30] and Qu et al. [31] reported a pronounced decrease in the final moisture content of Asian dry-fermented sausages, linked to the increase in the concentration of sugar used. The weight losses values obtained in this experiment were in line with those previously reported for similar products and ripening conditions [12,17]. In any case, the treatment and drying time applied made it possible to achieve an adequate a_W_ to inhibit the growth of microorganisms while at the same time ensuring a progressive reduction, since it should be considered that a rapid reduction in a_W_ can also slow down chemical and biochemical transformations in the meat mass or promote crusting phenomena in the sausage.

### 4.2. Instrumental Colour

Although the development of the internal colour of dry-fermented sausages is determined fundamentally by the meat fraction (lean and fat), it can often be affected by the spices and colorants used in the formulation, such as paprika or cochineal carmine (which increase the reddish or pinkish colour). In this work, neither colourants nor spices were used, to avoid masking the development of colour due to the ripening process. The values observed for the different colour coordinates were similar to those previously described for the meat product from this particular breed of pig (ranging between 38.80–45.30 for L* [25], 7.20–12.30 for a* and 4.90–7.60 for b* [32]).

The decrease observed in the L* values during the ripening process must be related to the dehydration of the sausage [28]. Although there were no significant differences due to the ripening time in all the batches, an increase in the a* value was observed in all the samples on days 14 and 21, followed by a decrease during ripening. This corresponds to the formation of nitrosylmyoglobin [33]. This pattern was not observed in batch C− due to the absence of added carbohydrates among its ingredients. A lower sugar concentration cannot favour the growth of coagulase-negative cocci (CNC), which promote the reduction of the nitrate/nitrite phenomenon [34]. The b* index is related to the fat content of the sausage; the decrease in its value during the ripening period has been previously described [35]. Moreover, the positive correlation found between the a* and b* parameters has already been observed by Álvarez et al. [36]. 

### 4.3. Texture Profile Analysis 

The results obtained were slightly below those reported by Hospital et al. [37] for dry-fermented sausages in terms of hardness and springiness but were similar for adhesiveness. During ripening, the drying of dry-fermented sausages is a crucial stage that affects their binding and rheological properties [13]. However, it also depends on other factors, such as nitrite content or the intensity of proteolysis, mediated by lactic acid bacteria that also induce a drop in pH. In our study, it was observed that the sausages with lower acidity (batch C−) showed lower values of hardness and springiness. This is consistent with findings by Herrero et al. [38], who highlighted the significant role of pH and a_W_ on these two variables (hardness and springiness) based on multivariate analysis.

The hardness of dry-fermented sausages depends on a balance between moisture loss and proteolysis. The decrease in pH leads to protein denaturation, facilitating the hydrolysis of proteins by LAB and CNC [25]. In batch C−, the pH remained at higher levels, resulting in less severe protein denaturation. This better preserved the WHC of the proteins in batch C−, reflected in lower hardness values.

Ordóñez et al. [39] have observed correlations between instrumental hardness and chewiness with sensory attributes. However, in our study, no correlations were found between these variables and sensory analysis results (See Table S1 from the Supplementary Materials). Nevertheless, chewiness was affected by carbohydrate content, as reported by Gonzalez et al. [13] in dry-fermented sausages with similar characteristics.

### 4.4. Sensory Analysis

The different carbohydrates used at the levels set in our study did not affect the sensory characteristics of the products. This is consistent with the observations by Hospital et al. [37], who worked with a similar formula to that of the present study: sausages made with 2% bee product mixtures, primarily composed of honey. No correlations were observed between sensory attributes and colour or TPA. Only texture and overall score showed a trend toward significance (*p* < 0.1) in their positive correlation with instrumental adhesiveness (r = 0.7213 and r = 0.7185, respectively).

## 5. Conclusions

Based on the results obtained, we can conclude that all the dry-fermented sausages examined in this study presented the expected instrumental and sensory characteristics throughout and by the end of the ripening process, regardless of the carbohydrate formula used in their manufacture. The slight differences detected between carbohydrate formulas should be studied in detail to establish more consistent conclusions. However, at first glance, sausages with a mixture of dextrose (in a higher proportion) and dextrin stood out from the others in terms of pH and instrumental colour, also standing out for their high ratings in sensory texture, which was closely related to the overall sensory score.

## Figures and Tables

**Table 1:** pH and aw evolution during ripening in dry-fermented sausages manufactured with different carbohydrate formulas.

	T	*n*	0	1	2	4	7	14	21	28	D	B	D × B
pH	C−	6	6.07 ± 0.04 ^a^	6.09 ± 0.01 ^a^	5.99 ± 0.04 ^a^	5.95 ± 0.00 ^a^	5.35 ± 0.04 ^b, A^	5.34 ± 0.04 ^b^	5.80 ± 0.05 ^a,A^	5.58 ± 0.01 ^b,A^	***	***	***
	D15	6	6.06 ± 0.00 ^a^	6.14 ± 0.01 ^a^	6.08 ± 0.02 ^a^	5.99 ± 0.03 ^a^	5.05 ± 0.04 ^b,B^	5.12 ± 0.26 ^b^	5.31 ± 0.26 ^b,BC^	5.15 ± 0.08 ^b,B^			
	D10d5	6	6.09 ± 0.01 ^a^	6.09 ± 0.01 ^a^	6.05 ± 0.04 ^a^	5.92 ± 0.05 ^a^	4.98 ± 0.06 ^d,B^	5.30 ± 0.10 ^bc^	5.43 ± 0.07 ^b,B^	5.10 ± 0.03 ^cd,B^			
	D5d10	6	6.10 ± 0.00 ^a^	6.07 ± 0.02 ^a^	6.00 ± 0.02 ^a^	5.90 ± 0.00 ^a^	5.02 ± 0.02 ^b,B^	5.09 ± 0.02 ^b^	5.28 ± 0.02 ^b,BC^	5.04 ± 0.14 ^b,B^			
	D5d5g5	6	6.03 ± 0.00 ^a^	6.02 ± 0.02 ^a^	6.02 ± 0.00 ^a^	5.93 ± 0.02 ^a^	5.01 ± 0.00 ^b,B^	5.11 ± 0.02 ^b^	5.04 ± 0.12 ^b,C^	5.10 ± 0.02 ^b,B^			
	d15	6	6.00 ± 0.00 ^a^	6.10 ± 0.02 ^a^	6.02 ± 0.00 ^a^	5.92 ± 0.01 ^a^	5.05 ± 0.01 ^b,B^	5.20 ± 0.03 ^b^	5.23 ± 0.06 ^b,BC^	5.11 ± 0.01 ^b,B^			
a_w_	C−	6	0.975 ± 0.005 ^a^	0.972 ± 0.001 ^a^	0.961 ± 0.003 ^ab^	0.964 ± 0.001 ^ab^	0.949 ± 0.002 ^b^	0.921 ± 0.003 ^c,AB^	0.909 ± 0.001 ^c^	0.877 ± 0.004 ^d^	***	*	***
	D15	6	0.986 ± 0.001 ^a^	0.971 ± 0.012 ^ab^	0.961 ± 0.006 ^b^	0.961 ± 0.005 ^b^	0.938 ± 0.003 ^c^	0.902 ± 0.001 ^d,D^	0.900 ± 0.005 ^d^	0.878 ± 0.005 ^e^			
	D10d5	6	0.979 ± 0.001 ^a^	0.967 ± 0.001 ^ab^	0.968 ± 0.001 ^ab^	0.956 ± 0.004 ^bc^	0.948 ± 0.007 ^c^	0.912 ± 0.001 ^d,BCD^	0.903 ± 0.000 ^d^	0.880 ± 0.007 ^e^			
	D5d10	6	0.977 ± 0.003 ^a^	0.968 ± 0.006 ^a^	0.963 ± 0.002 ^a^	0.965 ± 0.006 ^a^	0.945 ± 0.004 ^b^	0.926 ± 0.001 ^c,AB^	0.902 ± 0.013 ^d^	0.884 ± 0.002 ^e^			
	D5d5g5	6	0.980 ± 0.006 ^a^	0.966 ± 0.004 ^a^	0.960 ± 0.001 ^ab^	0.961 ± 0.002 ^ab^	0.944 ± 0.005 ^b^	0.929 ± 0.001 ^c,A^	0.903 ± 0.008 ^d^	0.885 ± 0.004 ^e^			
	d15	6	0.974 ± 0.001 ^a^	0.971 ± 0.004 ^a^	0.960 ± 0.001 ^a^	0.963 ± 0.000 ^a^	0.940 ± 0.002 ^b^	0.910 ± 0.007 ^c,CD^	0.901 ± 0.001 ^c^	0.885 ± 0.004 ^d^			

* *p* < 0.05; *** *p* < 0.001. ^a–e^ Means with different superscript in the same row indicate that the samples presented significant differences. ^A–D^ Means with different superscript in the same column indicate that the samples presented significant differences. T = Treatment, D = Day, B = Batch, D × B = interaction. C− = negative control, D15 = 15 g/kg dextrose, D10d5 = 10 g/kg dextrose + 5 g/kg dextrin, D5d10 = 5 g/kg dextrose + 10 g/kg dextrin, D5d5g5 = 5 g/kg dextrose + 5 g/kg dextrin + 5 g/kg glucose syrup, d15 = 15 g/kg dextrin.

**Table 2:** Evolution of moisture and weight-loss values during ripening in dry-fermented sausages manufactured with different carbohydrate formulas.

	T	*n*	1	7	14	21	28	D	B	D × B
Moisture (%)	C−	6	57.49 ± 2.27 ^a^	41.76 ± 0.75 ^bc^	39.68 ± 0.38 ^cd^	34.77 ± 1.38 ^cd^	29.05 ± 1.85 ^d^	***	ns	ns
	D15	6	56.97 ± 0.60 ^a^	40.70 ± 0.38 ^b^	33.76 ± 1.35 ^bc^	33.28 ± 2.52 ^bc^	29.78 ± 3.48 ^c^			
	D10d5	6	56.49 ± 2.69 ^a^	44.45 ± 1.75 ^b^	36.89 ± 0.29 ^bc^	32.65 ± 1.13 ^bc^	28.49 ± 0.61 ^c^			
	D5d10	6	57.04 ± 3.36 ^a^	45.36 ± 1.25 ^b^	37.39 ± 4.26 ^bc^	31.15 ± 3.78 ^cd^	28.63 ± 1.20 ^d^			
	D5d5g5	6	59.16 ± 4.85 ^a^	45.45 ± 2.13 ^b^	36.97 ± 2.77 ^bc^	32.48 ± 0.88 ^c^	28.60 ± 0.18 ^c^			
	d15	6	57.65 ± 5.24 ^a^	41.50 ± 1.69 ^b^	37.31 ± 0.77 ^bc^	35.04 ± 1.26 ^bc^	30.11 ± 0.72 ^c^			
Weight loss (%)	C−	6	1.76 ± 0.13 ^d^	21.14 ± 0.14 ^c,B^	29.39 ± 0.11 ^b^	33.74 ± 0.43 ^b^	38.85 ± 0.23 ^a^	***	**	ns
	D15	6	1.84 ± 0.52 ^d^	25.21 ± 1.39 ^c,A^	31.45 ± 0.70 ^b^	35.87 ± 0.02 ^ab^	38.45 ± 1.64 ^a^			
	D10d5	6	2.00 ± 0.19 ^d^	21.05 ± 0.09 ^c,B^	31.49 ± 0.40 ^b^	35.76 ± 0.26 ^ab^	38.43 ± 0.38 ^a^			
	D5d10	6	1.78 ± 0.10 ^d^	21.51 ± 0.51 ^c,AB^	31.44 ± 1.24 ^b^	35.31 ± 0.26 ^ab^	38.20 ± 1.30 ^a^			
	D5d5g5	6	1.79 ± 0.03 ^d^	22.28 ± 0.63 ^c,AB^	30.56 ± 0.87 ^b^	34.95 ± 0.55 ^b^	41.57 ± 4.41 ^a^			
	d15	6	2.20 ± 0.41 ^d^	24.52 ± 0.28 ^c,AB^	32.08 ± 1.81 ^b^	37.06 ± 1.23 ^a^	39.10 ± 1.11 ^a^			

ns: not significant; ** *p* < 0.01; *** *p* < 0.001. ^a–d^ Means with different superscript in the same row indicate that the samples presented significant differences. ^A,B^ Means with different superscript in the same column indicate that the samples presented significant differences. T = Treatment, D = Day, B = Batch, D × B = interaction. C− = negative control, D15 = 15 g/kg dextrose, D10d5 = 10 g/kg dextrose + 5 g/kg dextrin, D5d10 = 5 g/kg dextrose + 10 g/kg dextrin, D5d5g5 = 5 g/kg dextrose + 5 g/kg dextrin + 5 g/kg glucose syrup, d15 = 15 g/kg dextrin.

**Table 3:** Evolution of CIE L*a*b* parameters of dry-fermented sausages manufactured with different carbohydrate formulas and ripened for 28 days.

T	*n*	Day 1	Day 7	Day 14	Day 21	Day 28	D	B	D × B
				Lightness (L*)					
C−	6	40.05 ± 0.87 ^B^	41.60 ± 4.69 ^B^	41.09 ± 4.69	42.10 ± 1.94	43.99 ± 2.19 ^A^			
D15	6	42.38 ± 5.27 ^ab,AB^	41.09 ± 0.75 ^ab,B^	47.96 ± 2.70 ^a^	39.99 ± 1.51 ^b^	37.63 ± 3.52 ^b,B^			
D10d5	6	48.47 ± 2.68 ^a,A^	46.23 ± 0.94 ^ab,AB^	41.07 ± 0.13 ^abc^	38.87 ± 0.79 ^c^	36.89 ± 1.95 ^c,B^	***	***	***
D5d10	6	43.04 ± 4.64 ^AB^	43.45 ± 0.47 ^AB^	44.47 ± 2.52	43.48 ± 3.26	42.44 ± 0.16 ^AB^			
D5d5g5	6	44.61 ± 3.35 ^AB^	48.46 ± 0.49 ^A^	42.89 ± 2.10	43.41 ± 0.01	42.50 ± 1.32 ^AB^			
d15	6	45.73 ± 1.83 ^AB^	44.64 ± 1.76 ^AB^	45.89 ± 3.79	41.50 ± 4.46	42.50 ± 0.88 ^AB^			
				Redness (a*)					
C−	6	14.14 ± 0.07	13.84 ± 1.14	14.21 ± 1.96	13.91 ± 2.62	13.38 ± 3.70 ^A^			
D15	6	14.02 ± 3.26	14.75 ± 0.80	12.99 ± 0.44	14.09 ± 0.15	12.18 ± 0.63 ^AB^			
D10d5	6	11.47 ± 0.66 ^ab^	13.00 ± 1.55 ^ab^	13.37 ± 2.16 ^a^	12.59 ± 1.98 ^ab^	9.95 ± 0.30 ^b,B^	***	***	**
D5d10	6	11.85 ± 1.48 ^ab^	12.21 ± 2.21 ^ab^	13.08 ± 1.73 ^ab^	14.94 ± 3.19 ^a^	9.45 ± 0.76 ^b,B^			
D5d5g5	6	13.23 ± 4.24	11.73 ± 2.78	13.41 ± 2.09	11.92 ± 0.32	10.65 ± 0.03 ^AB^			
d15	6	13.37 ± 2.47	11.97 ± 0.41	12.46 ± 1.18	12.02 ± 2.54	12.74 ± 0.39 ^AB^			
				Yellowness (b*)					
C−	6	7.41 ± 1.34	7.10 ± 1.28 ^A^	5.59 ± 0.35	6.15 ± 2.56 ^A^	5.41 ± 1.55 ^A^			
D15	6	9.07 ± 0.27 ^a^	6.14 ± 0.35 ^ab,AB^	5.89 ± 0.26^b^	4.58 ± 0.10 ^bc,AB^	2.85 ± 0.79 ^c,B^			
D10d5	6	9.30 ± 1.79 ^a^	5.71 ± 0.36 ^b,AB^	4.30 ± 1.38 ^bc^	3.98 ± 1.48 ^bc,AB^	3.18 ± 0.69 ^c,AB^	***	**	***
D5d10	6	7.45 ± 1.42 ^a^	4.79 ± 1.73 ^ab,AB^	4.99 ± 0.10 ^ab^	5.68 ± 0.93 ^a,AB^	2.84 ± 0.18 ^b,B^			
D5d5g5	6	8.28 ± 1.50 ^a^	6.06 ± 2.56 ^ab,AB^	5.65 ± 0.94 ^bc^	3.36 ± 0.31 ^c,B^	3.59 ± 0.42 ^c,AB^			
D15	6	9.80 ± 1.82 ^a^	4.56 ± 0.19 ^b,B^	4.91 ± 2.36 ^b^	4.11 ± 0.98 ^b,AB^	4.23 ± 0.03 ^b,AB^			

** *p* < 0.01; *** *p* < 0.001. ^a–c^ Means with different superscript in the same row indicate that the samples presented significant differences. ^A,B^ Means with different superscript in the same column indicate that the samples presented significant differences. T = Treatment, D = Day, B = Batch, D × B = interaction. C− = negative control, D15 = 15 g/kg dextrose, D10d5 = 10 g/kg dextrose + 5 g/kg dextrin, D5d10 = 5 g/kg dextrose + 10 g/kg dextrin, D5d5g5 = 5 g/kg dextrose + 5 g/kg dextrin + 5 g/kg glucose syrup, d15 = 15 g/kg dextrin.

**Table 4:** Effect of carbohydrate formulas used on the instrumental texture parameters of dry-fermented sausages.

	*n*	C−	D15	D10d5	D5d10	D5d5g5	d15	B
Hardness (N)	6	19.4 ± 4.5 ^b^	30.6 ± 7.7 ^a^	28.1 ± 5.6 ^a^	29.6 ± 6.8 ^a^	27.2 ± 6.2 ^a^	26.9 ± 6.6 ^a^	**
Springiness	6	0.45 ± 0.09 ^b^	0.53 ± 0.09 ^ab^	0.56 ± 0.10 ^a^	0.52 ± 0.08 ^ab^	0.55 ± 0.09 ^a^	0.49 ± 0.08 ^ab^	*
Adhesiveness (N × s)	6	−0.67 ± 0.27	−0.82 ± 0.22	−0.72 ± 0.36	−1.64 ± 0.25	−1.17 ± 0.83	−0.78 ± 0.16	ns
Cohesiveness	6	0.69 ± 0.05	0.66 ± 0.08	0.69 ± 0.05	0.63 ± 0.08	0.70 ± 0.06	0.70 ± 0.06	ns
Chewiness (N)	6	6.2 ± 2.2 ^b^	10.9 ± 3.7 ^a^	10.8 ± 2.4 ^a^	10.1 ± 3.9 ^a^	10.4 ± 2.9 ^a^	9.3 ± 2.9 ^a^	**

ns: no significant; * *p* < 0.05; ** *p* < 0.01. ^a–b^ Means with different superscript in the same row indicate that the samples presented significant differences. C− = negative control, D15 = 15 g/kg dextrose, D10d5 = 10 g/kg dextrose + 5 g/kg dextrin, D5d10 = 5 g/kg dextrose + 10 g/kg dextrin, D5d5g5 = 5 g/kg dextrose + 5 g/kg dextrin + 5 g/kg glucose syrup, d15 = 15 g/kg dextrin, B = Batch.

## Data Availability

The original contributions presented in this study are included in the article/Supplementary Material. Further inquiries can be directed to the corresponding author.

## Supplementary Materials

The following supporting information can be downloaded at: https://www.mdpi.com/article/10.3390/foods14020248/s1, Figure S1: pH evolution during ripening in dry-fermented sausages manufactured with different carbohydrate formulas; Figure S2; Water activity during ripening in dry-fermented sausages manufactured with different carbohydrate formulas; Figure S3: Moisture and weight loss evolution during ripening in dry-fermented sausages manufactured with different carbohydrate formulas; Table S1: Pearson correlation coefficients between the different variables analyzed in the dry-fermented sausages.
