# Supplementary material for: Effect of Carbohydrate Formulas on Instrumental and Sensory Parameters in Dry-Fermented Iberian Pork Sausages
Source: Foods. 2025 Jan 14;14(2):248. doi: 10.3390/foods14020248 (PMC11764644; doi:10.3390/foods14020248)
Supplement: Supplementary file 1 [file foods-14-00248-s001.zip › foods-3356672-supplementary.pdf]

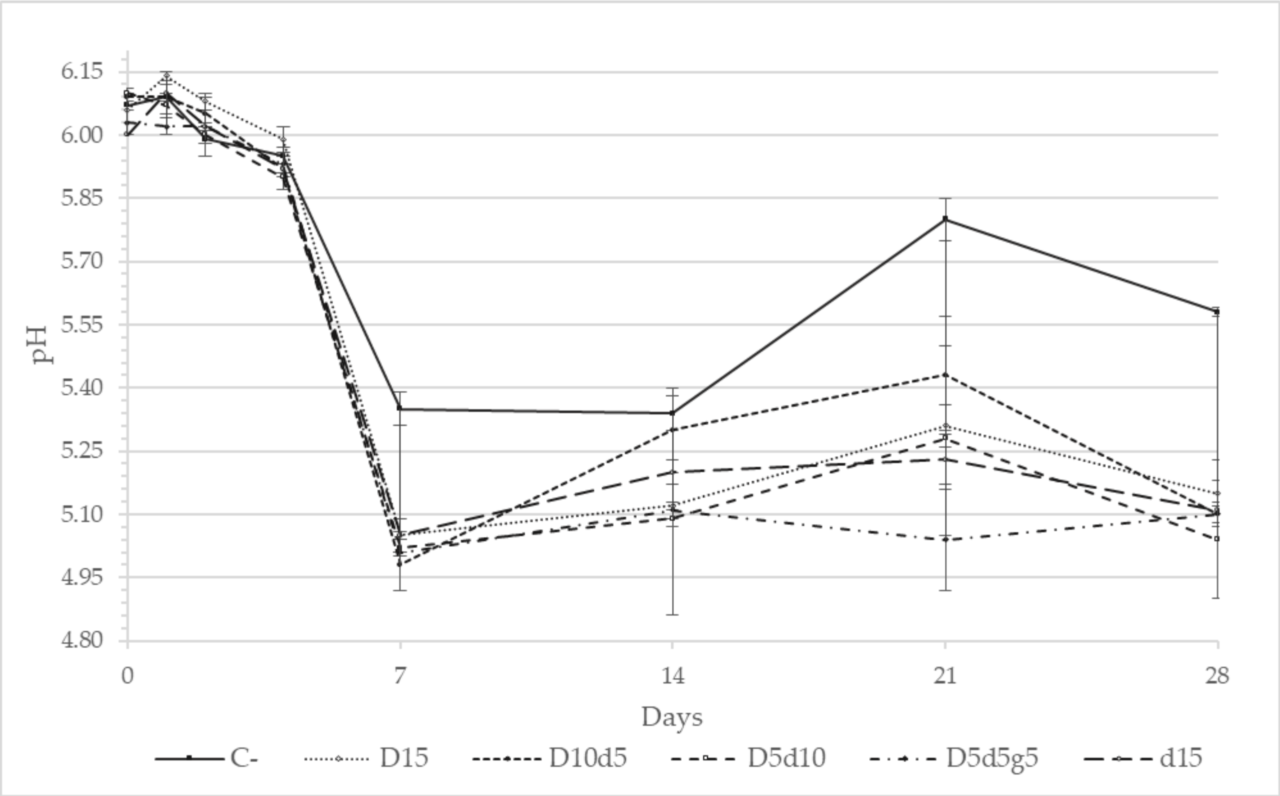

Figure S1. pH evolution during ripening in dry-fermented sausages manufactured with different carbohydrate formulas. C- = negative control, D15 = 15 g/kg dextrose, D10d5 = 10 g/kg dextrose + 5 g/kg dextrin, D5d10 = 5 g/kg dextrose + 10 g/kg dextrin, D5d5g5 = 5 g/kg dextrose + 5 g/kg dextrin + 5 g/kg glucose syrup, d15= 15 g/kg dextrin.

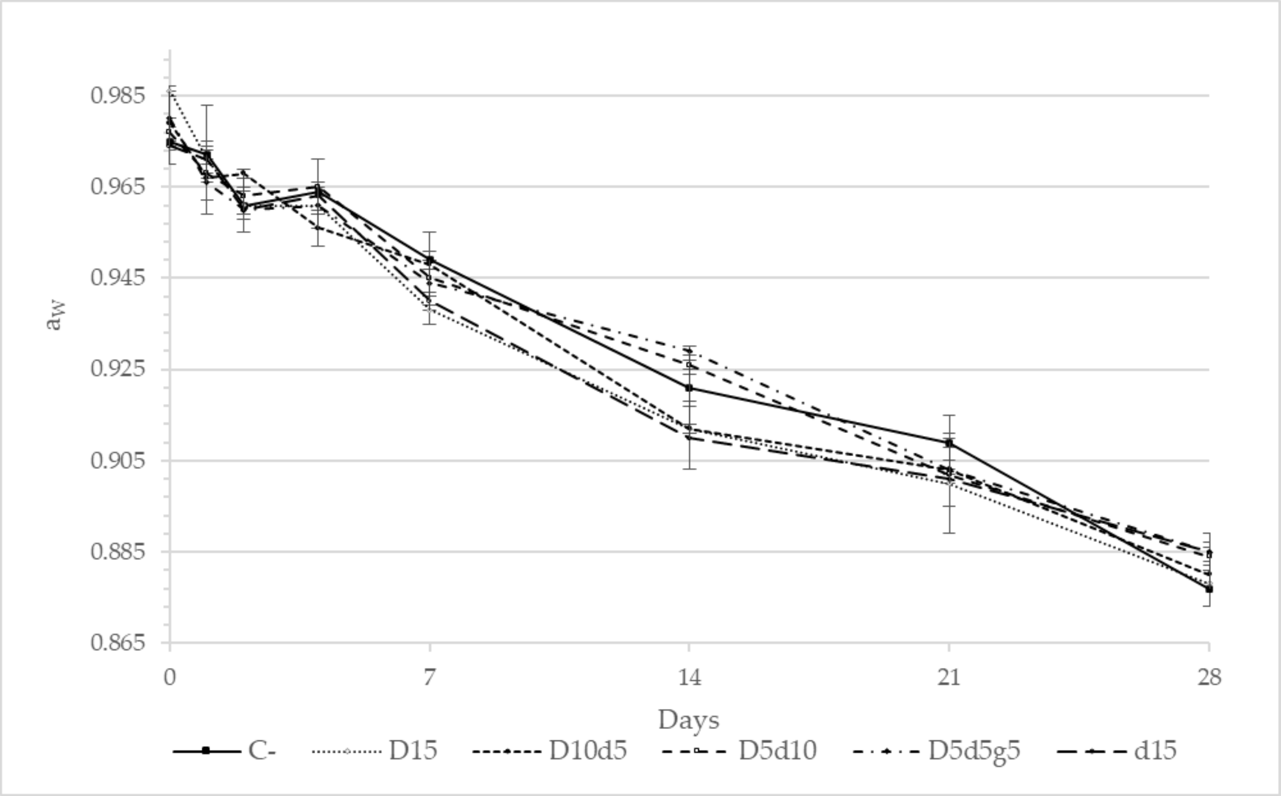

Figure S2. Water activity during ripening in dry-fermented sausages manufactured with different carbohydrate formulas. C- = negative control, D15 = 15 g/kg dextrose, D10d5 = 10 g/kg dextrose + 5 g/kg dextrin, D5d10 = 5 g/kg dextrose + 10 g/kg dextrin, D5d5g5 =5 g/kg dextrose + 5 g/kg dextrin+ 5 g/kg glucose syrup, d15= 15 g/kg dextrin.

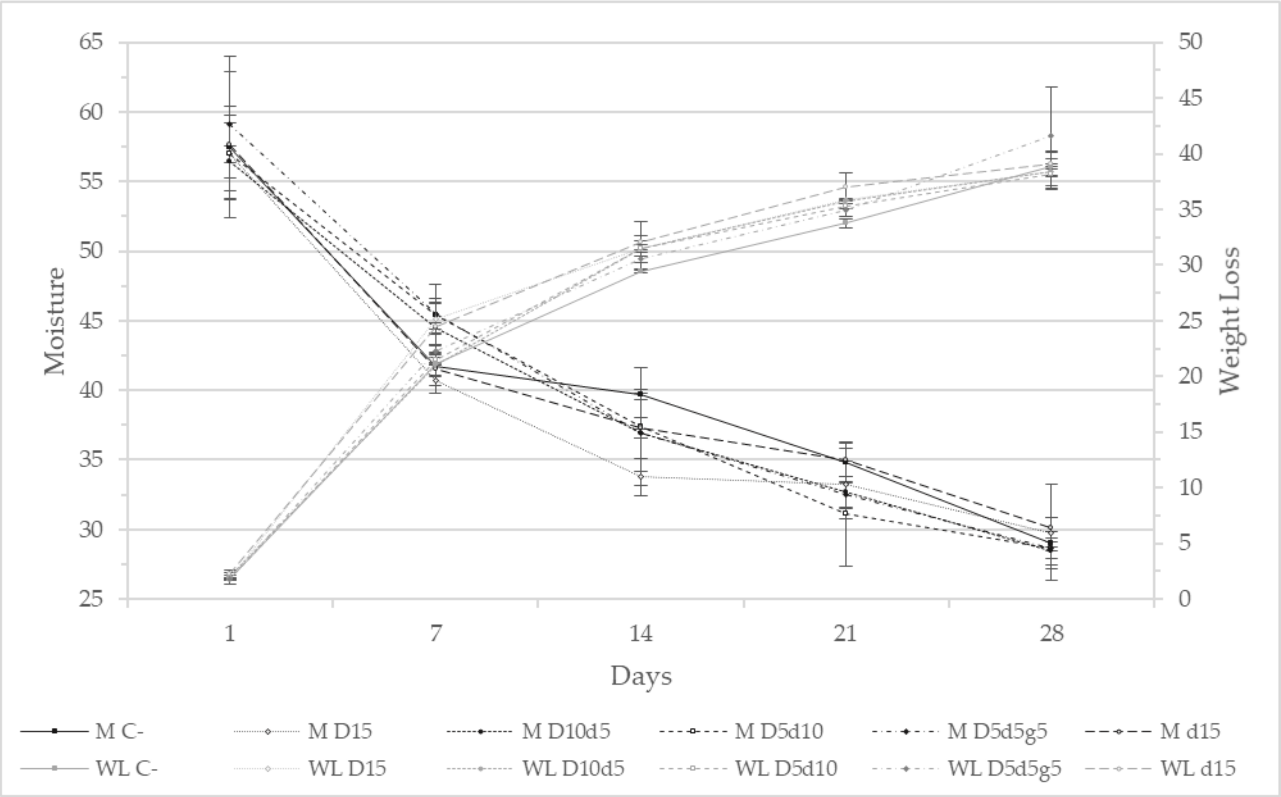

Figure S3. Moisture and weight loss evolution during ripening in dry-fermented sausages manufactured with different carbohydrate formulas. C- = negative control, D15 = 15 g/kg dextrose, D10d5 = 10 g/kg dextrose + 5 g/kg dextrin, D5d10 = 5 g/kg dextrose + 10 g/kg dextrin, D5d5g5 = 5 g/kg dextrose + 5 g/kg dextrin+ 5 g/kg glucose syrup, d15= 15 g/kg dextrin.

Table S1. Pearson correlation coefficients between the different variables analyzed in the dry-fermented sausages.

	**Lightness (L*)**	**Redness (a*)**	**Yellowness (b*)**	**Hardness (N)**	**Springiness**	**Adhesiveness (N*s)**	**Cohesiveness**	**Chewiness (N)**	**Lean Colour**	**Fat Colour**	**Texture**	**Overall Score**
**Lightness (L*)**	1.0000	-0.6909	-0.3465	0.0392	0.4337	-0.0307	0.3957	0.3061	-0.2762	-0.6077	-0.2095	-0.1059
**Redness (a*)**		1.0000	0.7489	-0.4918	-0.7882	0.5803	0.2460	-0.6333	0.3311	0.6707	0.5790	0.4218
**Yellowness (b*)**			1.0000	**-0.8715**	-0.7909	0.3429	0.2998	**-0.9059**	-0.1929	0.0831	0.2587	0.1491
**Hardness (N)**				1.0000	0.6130	-0.4756	-0.4553	0.8881	0.0674	-0.0068	-0.3484	-0.3276
**Springiness**					1.0000	-0.3482	-0.0002	**0.8712**	0.0388	-0.3139	-0.1520	-0.0097
**Adhesiveness (N*s)**						1.0000	0.7671	-0.3082	0.7367	0.6938	**0.9305**	**0.8986**
**Cohesiveness**							1.0000	-0.0946	0.3229	0.1690	0.7213	0.7185
**Chewiness (N)**								1.0000	0.1497	-0.0906	-0.1437	-0.0694
**Lean Colour**									1.0000	**0.8918**	**0.8293**	**0.8495**
**Fat Colour**										1.0000	0.7753	0.7105
**Texture**											1.0000	**0.9793**
**Overall Score**												1.0000
